# Prevalence of rotavirus infection among children with acute diarrhoea after rotavirus vaccine introduction in Kenya, a hospital cross-sectional study

**DOI:** 10.1186/s12887-018-1291-8

**Published:** 2018-10-11

**Authors:** Catherine Muendo, Ahmed Laving, Rashmi Kumar, Boniface Osano, Thaddaeus Egondi, Pamela Njuguna

**Affiliations:** 1 P.O.Box 12487–00400, Nairobi, Kenya; 2 Department of Paediatrics and Child Health, University of Nairobi, P.O. Box 19676–00202, Nairobi, Kenya; 3 Drugs for Neglected Diseases initiative, P.O. Box 21936–00505, Nairobi, Kenya; 4 Public Health Specialist, Afya Resource Associates, P. O. Box 238–00202, Nairobi, Kenya

**Keywords:** Rotavirus associated diarrhoea, Children, Rotavirus vaccine, Kenya

## Abstract

**Background:**

Rotavirus infection is the most common cause of acute gastroenteritis globally in children under 5 years of age and is responsible for approximately 5% of all child deaths yearly. Rotavirus vaccination is considered an effective public health strategy to prevent infection and reduce the severity of disease. Multi-centre country trials on rotavirus vaccines demonstrated efficacy rates of more than 85% in developed countries but only about 65% in developing nations. Rotavirus vaccination was introduced into the Kenya Expanded Programme on Immunization (KEPI) in 2014. The objective of our study was to determine the prevalence of rotavirus infection, severity of acute diarrhoea and to determine the rotavirus vaccination status among children aged 3–24 months presenting with acute diarrhoea at Kenyatta National Hospital after introduction of rotavirus vaccine in Kenya.

**Methods:**

A total of 365 children aged 3–24 months presenting with acute diarrhoea at KNH were recruited from August 2016 to April 2017. Data on rotavirus vaccination status, nutritional status, feeding practices and sociodemographic characteristics were obtained and a full clinical evaluation of the patients was done. Severity of the gastroenteritis was assessed using the 20 point Vesikari Clinical Severity Scoring System. The children who were admitted were followed up for 7 days using hospital ward registers. Comorbid conditions were established from patient’s clinical records and physical examination. Stool specimens from study participants were tested for rotavirus using a commercially available enzyme linked immunosorbent immunoassay kit- ProSpecT Rotavirus Microplate Assay.

**Results:**

Majority of the children (96.7%) had received rotavirus vaccinations. The overall rotavirus prevalence was 14.5% and was higher among 17–24 months at 19.5%. The prevalence somewhat differed by gender, nutritional status, exclusive breastfeeding status, age and education level of mother/caregiver. Overall, a half of the children had severe acute diarrhoea and there were some differences in severity by child/mother characteristics.

**Conclusion:**

There is still burden of rotavirus diarrhoea after introduction of rotavirus vaccine and the prevalence varies by child characteristics.

## Background

Diarrhoeal diseases remain a leading cause of morbidity and mortality among children in the world, more so in developing countries with rotavirus infection being the most common cause of severe, acute diarrhoea [1]. Globally, it was estimated to cause 527,000 deaths in the year 2008 among children below 5 years of age, [2] this has since reduced to 215,000 in the year 2015 in the same age group [1]. More than 80% of these deaths continue to occur in South Asia and Sub-Saharan Africa [3]. Early complimentary feeding, nutritional status, dehydration and age less than 2 years are important risk factors associated with rotavirus diarrhoea. [4–6]. The peak infection age range with rotavirus is 3–24 months, the highest rate being between the ages of 6–11 months [7].The reported prevalence of rotavirus diarrhoea among children below 5 years hospitalized with diarrhoea from global surveillance networks and hospital based studies varies greatly ranging from 6 to 56% [8, 9]. In Kenya, the rotavirus prevalence was 40% among children below 5 years of age hospitalized for treatment of acute gastroenteritis [9] in the period 2006 to 2008. The clinical presentation of rotavirus illness ranges from mild, watery diarrhoea to severe diarrhoea with vomiting and fever that can result in dehydration with shock, electrolyte imbalance, and even death [10]. The Vesikari clinical severity scoring system (VCSSS) has been used in clinical trials in assessing rotavirus vaccine efficacy and effectiveness as a tool for defining the primary end point, which is severe rotavirus gastroenteritis [11]. It has also been used in clinical studies as a measure of acute gastroenteritis severity [12–14]. The parameters and categories of this severity scale are shown in Table 1.

**Table 1:** Vesikari Clinical Severity Scoring Scale

		SCORE	
PARAMETER	1	2	3
Diarrhoea			
Maximum number of stools per day	1–3	4–5	≥6
Diarrhoea duration (days)	1–4	5	≥6
Vomiting			
Maximum vomiting episodes per Day	1	2–4	≥5
Vomiting duration (days)	1	2	≥3
Temperature (°C)	37.1–38.4	38.5–38.9	≥39.0
Dehydration	None	Some	Severe
Treatment	Rehydration	Hospitalization	N/A
Severity scoring scale	< 7(mild)	7–10(moderate)	≥11(severe)

Vaccination has been shown to be the best way to prevent severe rotavirus disease [15, 16]. Currently available rotavirus vaccines have been shown to be effective in reducing the rotavirus disease burden with observed efficacy rates of about 65% in developing countries in Africa [15]. Rotavirus vaccines have been included in most national immunization programs in the world to date. However, it was not until July 2014, that the rotavirus vaccine was incorporated into the Kenya Expanded Program of Immunization (KEPI) [17].

The rotavirus vaccines available in Kenya are Rotarix®, manufactured by GlaxoSmithKline, administered orally in a 2-dose schedule, currently issued countrywide under the KEPI and RotaTeq®, manufactured by Merck & Co. Inc. and administered orally in a 3-dose schedule, mostly in private facilities.

In South Africa, a decline was reported in rotavirus prevalence and hospitalisations among children below 5 years after introduction of rotavirus vaccine in 2009 [18]. Similarly, a Rotavirus Sentinel Surveillance performance feedback report by WHO in 2016 reported a significant decline of rotavirus infection among countries in East and Southern Africa from 44% in 2010 to 25% in 2015, after introduction of rotavirus vaccine from the year 2014 [19]. Though much is known about the morbidity and mortality of rotavirus diarrhoea before introduction of rotavirus vaccine, there has not been a study to determine the change in the clinical profile of children being treated with acute diarrhoea after the introduction of the rotavirus vaccine in Kenyatta National Hospital. Therefore, this study aimed to determine the prevalence of rotavirus diarrhoea and severity of acute diarrhoea among children aged 3–24 months at Kenyatta National Hospital after rotavirus vaccine introduction in Kenya and also to determine the rotavirus vaccination status among the children.

## Methods

This study used data from survey conducted from August 2016 to April 2017 during the paediatrics residency period of the lead author. The study was conducted in the paediatric emergency unit and wards of Kenyatta National Hospital, Kenya’s largest public teaching and referral hospital; situated in the capital city, Nairobi. The hospital serves the low and middle-income population from Nairobi and its environs as well as referrals from other hospitals in the country and the greater Eastern Africa region.

### Clinical methods

The study was conducted among children aged 3 to 24 months presenting with acute diarrhoea, which was defined as passage of three or more loose stools per day lasting less than 14 days. Sequential sampling of patients who met the inclusion criteria was done in the paediatric wards and the paediatric emergency unit, then informed written consent was obtained from the caretaker. We obtained data on rotavirus vaccination status, nutritional status (z-scores), feeding practices and sociodemographic characteristics such as age, gender and caretaker characteristics such as age, level of education and relationship with the child using a pre- structured questionnaire followed by a full clinical evaluation. Rotavirus vaccination status was verified from the mother baby booklet and/or word of mouth as reported by the caretakers. Caretakers who did not recall the names of the vaccines received, described the vaccine by route of administration and the age of the child when they received the particular vaccine. Both rotavirus and oral polio vaccines are administered orally, thus rotavirus vaccine was distinguished from the oral polio vaccine by parents who described the oral polio vaccine as 2 drops administered orally compared to rotavirus which was administered orally with a prefilled 1 ml syringe/vial and had a thicker consistency. The severity of the gastroenteritis was assessed using the 20-point Vesikari Clinical Severity Scoring System. Comorbid conditions were established from the patient’s clinical records and physical examination. The patients who were admitted were followed up for 7 days using hospital ward registers to determine the outcome as either discharged, died or still admitted after 7 days. The duration of admission (in days) from the paediatric emergency unit was recorded.

### Laboratory measurements

The collected stool samples were transported within 5 min of sample collection to a centrally placed refrigerator found in the paediatric emergency unit and wards and stored at 2–8 °C. Thereafter, the stool samples were collected by a well-trained research assistant and transported twice daily to the Immunology laboratory-Kenyatta National Hospital using a cooler box that was maintained at a temperature of 2–8 °C. At the laboratory, the stool samples were frozen at − 20 °C prior to testing. They were tested for rotavirus antigen using a commercially available Enzyme-linked immunosorbent assay kit- ProSpecT Rotavirus Microplate Assay which is based on detection of group specific antigen in group A rotaviruses [20]. The test has a 95% sensitivity and specificity.

Rotavirus testing was carried out by a laboratory technologist trained in rotavirus detection using standardized operating procedures. The results were released and placed in the patient’s medical records.

### Control of bias and errors

The questionnaire was pretested to reduce measurement bias, ensuring the questions are sensitive enough to detect the variable of interest. Additionally, the research assistants were trained on a standardised data collection procedure and the equipment used such as the digital thermometers, digital infant scale and balance beam were inspected daily to ensure correct data measurements.

### Statistical analysis

A sample size of 365 children aged 3–24 months was available for analysis. The sample size of 365 and observed prevalence of 14.5% guarantees a power of 86% in estimating prevalence with a precision of 5% with 95% confidence level. The power calculation was performed using STATA command *sampsi*. The prevalence of rotavirus was estimated and summarized by the child and parent/caregiver characteristics. The distribution of Vesikari clinical severity score was compared between children who tested positive for rotavirus versus those tested negative using boxplot. The Vesikari clinical severity score was grouped into mild, moderate and severe which was summarized by the child and parent/caregiver characteristics in terms of frequencies and proportions. To assess the relationship of child and parent/caregiver characteristics by rotavirus infection was done using logistic regression. Vesikari clinical severity score was dichotomized into severe and none severe (mild and moderate). Then logistic regression was used to assess child and mother characteristics associated with severity of diarrhoea based on Vesikari. The clinical parameters of the Vesikari scoring for severe gastroenteritis was summarized for a subset of children who tested positive of rotavirus. All the analysis was performed using STATA version 15.

## Results

### Summary of recruited children

**Fig. 1:**
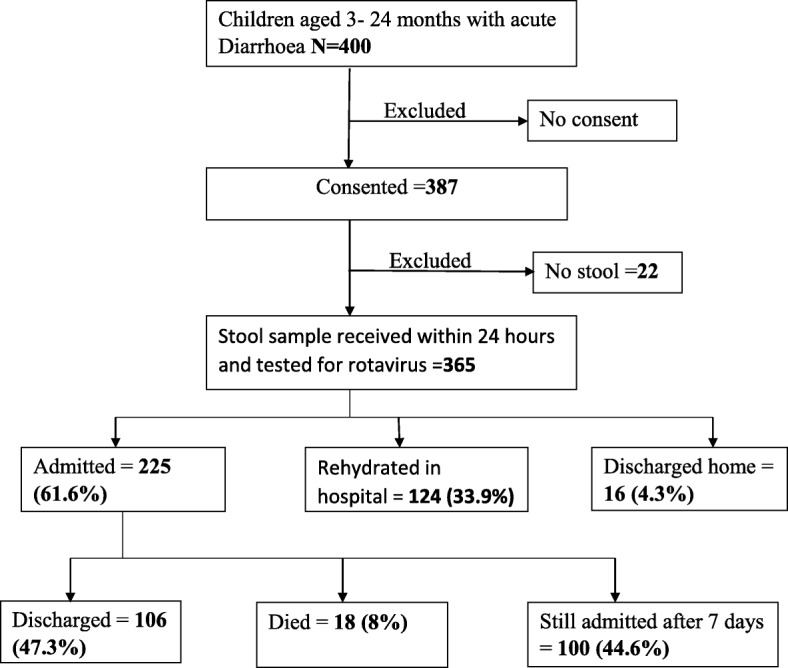
Flow of patients

**Fig. 2:**
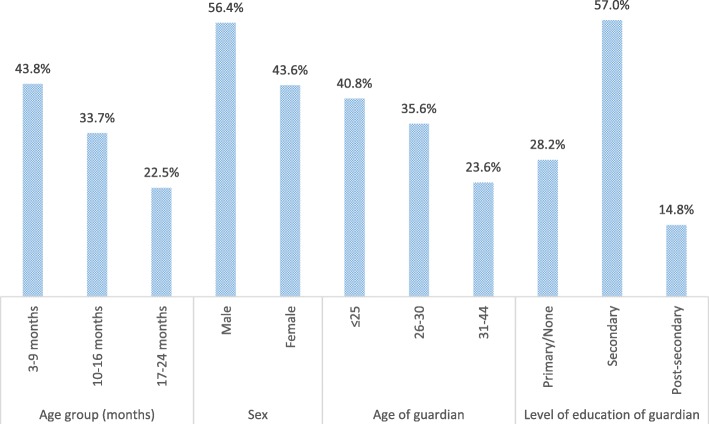
The distribution of sample children by child/caregiver characteristics

A total of 400 children aged 3–24 months with acute diarrhoea were seen at Kenyatta National Hospital for the period of August 2016 to April 2017 (Fig. 1). Thirteen (3.3%) children were excluded because no consent was provided and 22 (5.5%) had no stool sample. Therefore, a total of 365 (91.3%) children were included for analysis into the study. The median age of the children analysed was 11 months (IQR 7–16 months). The age group of 3–9 months old children formed majority of children at 43.8%. There were more male children (56.4%) than females. Exclusive breastfeeding was reported for 73.4% of the children while 68.5% were wasted (≤ − 2 SD). Most children (97.8%) were under the care of their mothers whose age ranged from 17 to 44 years with average age of 27.3 (SD = 4.74). The summary of caregiver/child characteristics are presented in Fig. 2.

### Prevalence of rotavirus

Rotavirus was detected in 53 children stool samples resulting in prevalence of 14.5% (95% CI 11.1–18.6). Table 2 provides the prevalence (percent of those positive) by child/mother characteristics. The observed prevalence was higher among children of older age group ranging from 10.6% for 3–9 months to 19.5% for 17–24 months. The prevalence was higher among male children (16.0% vs 12.6%). It was rather surprising that the observed prevalence was lower among wasted children compared to normal (12.8% vs 18.3%) and higher among exclusively breastfed children (16.0% vs 10.3%). The distribution of prevalence by mother/caregiver age showed somewhat increasing trend with a prevalence of 12.1% for young age group of below 25 years and 17.4% for older age group of 31–44 years. Mothers with primary or no education had higher prevalence and more severe disease compared to those with at least secondary level of education (18.4% vs 13.0%).

**Table 2:** Rotavirus prevalence and diarrhoea severity by child/caregiver characteristics

	Positive	mild	moderate	severe
	*n* (%)	*n* (%)	n (%)	*n* (%)
Age group (months)				
3–9 months	17 (10.6)	14 (8.8)	60 (37.5)	86 (53.8)
10–16 months	20 (16.3)	12 (9.8)	51 (41.5)	60 (48.8)
17–24 months	16 (19.5)	9 (11.0)	34 (41.5)	39 (47.6)
Sex				
Male	33 (16.0)	18 (8.7)	86 (41.7)	102 (49.5)
Female	20 (12.6)	17 (10.7)	59 (37.1)	83 (52.2)
Nutritional Status				
Normal (>-2SD)	21 (18.3)	4 (3.5)	33 (28.7)	78 (67.8)
Wasted (<-2SD)	32 (12.8)	31 (12.4)	112 (44.8)	107 (42.8)
Exclusive breastfeeding				
Yes	43 (16.0)	27 (10.1)	103 (38.4)	138 (51.5)
No	10 (10.3)	8 (8.2)	42 (43.3)	47 (48.5)
Age of guardian				
≤ 25	18 (12.1)	21 (14.1)	59 (39.6)	69 (46.3)
26–30	20 (15.4)	7 (5.4)	56 (43.1)	67 (51.5)
31–44	15 (17.4)	7 (8.1)	30 (34.9)	49 (57.0)
Level of education of guardian				
Primary/None	19 (18.4)	6 (5.8)	39 (37.9)	58 (56.3)
Secondary	27 (13.0)	25 (12.0)	84 (40.4)	99 (47.6)
Post-secondary	7 (13.0)	4 (7.4)	22 (40.7)	28 (51.9)
Total	53 (14.5)	35 (9.6)	145 (39.7)	185 (50.7)

### Severity of Diarrhoea

Table 2 also provides the distribution of diarrhoea severity with child/mother characteristics. The observed proportion of children with severe diarrhoea was lower for older children and almost similar among male and female children. Similar observation for rotavirus prevalence was made for diarrhoea severity with nutrition status, exclusive breastfeeding, age and education of mother or caregiver.

Fifty percent of the children had severe acute diarrhoea. Two hundred and twenty five children (61.6%) were admitted to the paediatric wards. Of those children admitted to the wards, 216(96.4%) children had associated comorbidities with the commonest comorbidity being pneumonia in 57% followed by meningitis in 29% of the children.

### Clinical severity among rotavirus infected children

Table 3 presents the Vesikari Clinical Severity score among rotavirus infected children; Twenty-one children (39.6%) were rated mild, 17 (32.1%) rated moderate while 15 (28.3%) rated severe.

**Table 3:** Distribution of cases within the clinical parameters of the Vesikari scoring for severe gastroenteritis *n* = 53

	*n* (%)
Diarrhoea Duration (Days)	
1–4	42(79.3)
5	5(9.4)
≥ 6	6(11.3)
Frequency of diarrhoea per day	
3	29(54.7)
4–5	19(35.9)
≥ 6	5(9.4)
Duration of vomiting (days)	
0	5(9.4)
1	22(41.5)
2	15(28.3)
≥ 3	11(20.8)
Frequency of vomiting per day	
0	5(9.4)
1	17(32.1)
2–4	24(45.3)
≥ 5	7(13.2)
Temperature (°C)	
37.1–38.4	18(33.9)
38.5–38.9	25(47.2)
≥ 39.0	10(18.9)
Dehydration status	
None	9(17)
Some dehydration	6(11.3)
Severe dehydration/shock	38(71.7)
Treatment	
Rehydrated	21(39.6)
Admitted	32(60.4)
Severity Category	
Mild	21(39.6)
Moderate	17(32.1)
Severe	15(28.3)

Majority of children with rotavirus were reported to have had diarrhoea for 1–4 days (79.3%) with a frequency of 3–5 episodes of diarrhoea per day (91.6%). Almost all children reported vomiting (91%) with a frequency of 1–4 vomiting episodes per day (80%). More than two-thirds of the children had severe dehydration/shock (71%). Additionally, 60 % of the children were admitted.

### Rotavirus vaccination status

Most children 353 (96.7%) had been fully vaccinated against rotavirus. They had received 2 doses of rotarix vaccine, 3(0.8%) children had received only one dose of rotarix vaccine, while 9(2.4%) children had not been vaccinated for rotavirus. The 53 positive rotavirus cases had been fully vaccinated against rotavirus while the 12 children who had received partial or no vaccination were rotavirus negative.

### Factors associated with rotavirus infection or severe diarrhoea

Logistic regression was used to assess significance of factors at child level (age, gender, nutrition status and exclusive breastfeeding) and mother/caregiver level (age and education level). Table 4 presents logistic regression results for both rotavirus infection and diarrhoea severity. There was no statistically significant association of rotavirus infection with any of the factors considered for the analysis. However, there seemed to be interesting patterns in the level of association observed. The risk of rotavirus infection increased with age of the child. Children aged 17–24 months were twice more likely to be infected than children aged 3–9 months while those aged 10–16 months were 1.5 times more likely. There was also some level of association, although weak, between rotavirus infection and duration of exclusive breastfeeding. The infants who were breastfed for 6 months or longer were 1.4 times at risk of having rotavirus infection. The risk of rotavirus infection seemed higher among older mothers and lower for mothers with higher level of education.

**Table 4:** Logistic regression of child/caregiver characteristics and rotavirus infection or severe diarrhoea

	Rotavirus Infection		Severe diarrhoea	
	Odds Ratio [95 CI]	*p*-value	Odds Ratio [95 CI]	*p-*value
Child age (ref: 3–9 months)				
10–16 months	1.52 [0.73–3.15]	0.265	0.72 [0.43–1.20]	0.202
17–24 months	2.00 [0.92–4.36]	0.080	0.75 [0.42–1.33]	0.319
Female (ref: Male)	0.70 [0.38–1.30]	0.263	1.13 [0.73–1.74]	0.578
Wasting	0.67 [0.36–1.24]	0.199	0.36 [0.22–0.58]	< 0.001
Exclusive Breastfeeding	1.38 [0.63–2.99]	0.418	1.21 [0.73–2.02]	0.462
Mother/caregiver age (ref: ≤25)				
26–30	1.25 [0.62–2.52]	0.532	1.29 [0.79–2.11]	0.315
31–44	1.41 [0.65–3.02]	0.382	1.52 [0.87–2.66]	0.141
Mother/caregiver education (ref: primary/none)				
Secondary	0.64 [0.33–1.24]	0.187	0.76 [0.46–1.25]	0.280
Post-secondary	0.59 [0.23–1.54]	0.282	0.75 [0.38–1.48]	0.404

Similarly, apart from wasting there was no statistically significant association of severe diarrhoea and the remaining factors. It was interesting that malnourished (wasted) children were 64% less likely to have severe diarrhoea.

## Discussion

In this study, the observed prevalence of rotavirus diarrhoea was 14.5% which was lower than what was observed by Osano in 2008 at 38.2% [10] and Karanja in 2009 at 39.5% [21]. These studies were conducted before rotavirus vaccine was introduced into the Kenya National Immunization Program (KEPI). A Rotavirus Sentinel Surveillance performance feedback report by World Health Organization reported a decline of rotavirus associated diarrhoea among countries in East and Southern Africa from 40% in 2014 to 25% in 2015, after introduction of rotavirus vaccine from the year 2014 [19] showing a reduction in the burden of rotavirus with the introduction of the rotavirus vaccine. Our result however, may not be a true reflection of rotavirus burden in the community because of lack of data on the prevalence of rotavirus diarrhoea before introduction of vaccine in similar setting. Nonetheless, in a community study in Nicaragua they observed a 40% lower incidence rate of watery diarrhoea episodes suggestive of a reduction in rotavirus infection in the vaccine period as compared with the pre-vaccine period. This reduction may be attributable to herd immunity that results from an overall protective effect of the immunization program on both immunized and non-immunized children [22]. Similarly, this study noted that the children who presented with acute diarrhoea and were not vaccinated did not have rotavirus positive stools; it is postulated that it may be as a result of the overall protective effect of the rotavirus vaccine on both the immunized and non-immunized children [22].

The Vesikari Clinical Severity scoring system for gastroenteritis used in this study, elicited the distribution of severe diarrhoea as similar among those children who were rotavirus positive compared to those who were negative. Comparability of severity of rotavirus diarrhoea with other studies is difficult due to varied classification systems, some studies have described severity of rotavirus associated diarrhoea using the vesikari clinical scoring system, while others have described severity using the hydration status [21, 23] or the need for hospitalization as a marker for severity of illness [10]. Gatinu’s study in 2007 reported a 47.9% prevalence of severe dehydration as a marker of severe rotavirus disease [23]. The hospitalization rate in our study was observed to be above 60%. This could be attributable to associated comorbidities as a majority of the children admitted had associated comorbidities that necessitated hospitalization, the commonest being pneumonia. Severe dehydration commonly presents as fast and deep acidotic breathing due to electrolyte imbalances and metabolic acidosis as a result of fluid loss and may be misdiagnosed as pneumonia due to similar presentation [24]. However, other studies demonstrate concurrent pneumonia infection in children presenting with diarrhoea [24]. The results indicate that the risk of rotavirus infection increased with age of the child which is quite contrary to most studies which report increased risk of rotavirus infection in infants [5, 10, 25]. According to the World Health Organization scientific working group, most cases of rotavirus infections occur in children between 6 and 24 months with a peak incidence at 9 to 12 months [7]. It is postulated that younger children tend to be at an increased risk of developing severe dehydration due to their small body size, as they lose a greater portion of their total fluid volume during the illness [26].

We found that the exclusively breastfed children were about one and a half more times likely to have rotavirus diarrhoea, though it was not statistically significant. It is thought that breastfeeding reduces gastrointestinal infections as breast milk contains secretory antibodies such as secretory IgA, immune cells and other defense factors such as lactoferrin, oligosaccharides and human milk glycans that protect the intestinal epithelium against pathogens [27]. However, the specific role of breastfeeding in the prevention of rotavirus diarrhoea has not been well established but it is generally considered to at least reduce the severity of the disease [28]. There have been conflicting results as to whether breastfeeding is protective or not. Naficy et al. found a lower incidence of rotavirus diarrhoea in infants that received breast milk [29] and others have shown evidence that breastfeeding offers protection against only severe rotavirus infections [28]. On the contrary, Gurwith and Totterdell found no evidence of protection against clinical rotavirus disease by maternal milk [30, 31]. The role of exclusive breastfeeding needs to be explored further in a study designed to establish whether exclusive breastfeeding protects against rotavirus diarrhoea. Malnourished children were found to have less risk of developing severe diarrhoea in our study. Interestingly, it is postulated that malnutrition is associated with protection from rotavirus diarrhoea for various reasons, among these, the possibility of shortening of villi in malnourished infants that may inhibit rotavirus entry and replication [32]. However, some studies show that nutritional status has no significant correlation with severity of rotavirus diarrhoea [33].

The rotavirus vaccination status among the children in this study was found to be at 96.7% against rotavirus. However, vaccination of the child was verified from the maternal and child booklet and/or word of mouth from the parent. For the parents who did not recall the names of the vaccines received, they described the vaccine by route of administration and the age of the child when they received the particular vaccine. The latter method of vaccine verification is unlikely to be as accurate as the written and dated records with a high likelihood of overreporting [34, 35]. The Kenya Demographic Health Survey 2014/2015 reported that 79% of children had received all the basic vaccinations [36]. There was no specific rotavirus vaccine coverage report in the Kenya Demographic Health Survey by 2014 as the vaccine had just been rolled out for use in the country in mid-2014 however, according to WHO/UNICEF the rotavirus vaccine achieved only 38% coverage in 50% of the national target Kenyan population in 2014 and 66% in 2015 [37]. Contributing factors associated with the high rotavirus vaccination status were not explored in this study.

Interpretation of results from this study should be done with caution because most results were not statistically significant which could be due to the sample size of the study.

## Conclusions

The burden of rotavirus associated diarrhoea among children aged 3–24 months at Kenyatta National Hospital in 2017 was observed to be 14.5%. The results provide the level of burden of rotavirus infection but are not able to conclude on the attributable effect of introduction of the rotavirus vaccine. Though not statistically significant, there seems to be some interesting pattern for both rotavirus infection and severity of diarrhea with child/parent characteristics. The rotavirus vaccination status was 96.7% among the children. Community based surveillance studies are needed to establish the prevalence of rotavirus at a population level and identify associated risk factors for infection.

## Abbreviations

CHERG: Child Health Epidemiology Reference Group
IQR: Interquartile range
KEPI: Kenya Expanded Program of Immunization
KNH: Kenyatta National Hospital
VCSSS: Vesikari clinical severity scoring system
